# Scottish Asylums

**Published:** 1910-10-08

**Authors:** 


### SCOTTISH ASYLUMS.

We gather from the recent report of the Scottish Lunacy Commissioners that on January 1, 1910, there were under supervision in Scotland 18,337 lunatics. Of these, 8,978 were males (7,823 pauper, 1,108 private), and 9,359 females (7,901 pauper, 1,452 private). The figures show an increase of 111 registered lunatics as compared with the number registered on January 1, 1909. This is the smallest increase recorded since the establishment of the Board. Private cases have decreased by 72, but pauper patients have increased by 183. The Criminal Lunatic Department of Perth Prison shows an increase of two cases in the year, while 27 more imbecile children were being educated in the training schools. During the year 215 private patients were discharged recovered, or 19 less than the year previous and 37 below the average for the five years 1900-1904. The number of pauper patients discharged recovered was 1,030, or 151 less than the year previous and 261 below the average for the same quinquennial period. The recoveries per cent. of admissions, excluding transfers, were respectively 43.4 for private and 37.4 for pauper cases, figures which show no falling-off as regards private patients, but a decrease among paupers. This falling-off in the proportion of recoveries among the poorer classes has been continuous since 1880, and the tendency still seems to be towards a further decrease. During the year 149 private patients were discharged unrecovered as well as 369 paupers, giving of discharges unrecovered (excluding transfers) of average number resident, percentages respectively of 6.4 and 3.0. Among private patients the number of deaths was 209, or 9 per cent. Among paupers 1,184 deaths occurred, or 9.5 per cent. The percentages for the previous year were respectively 7.3 and 9.3. The whole number of escapes from establishments during 1909 was 145. Of these, 68 were brought back within twenty-four hours, 44 within a week, and 11 after a week. The remaining 22 were still absent after 28 days. Of these, 5 were removed from the asylum registers as recovered, 5 as relieved, 9 as not improved, and 3 died. The escapes amounted to 9 per 1,000 of patients in establishments. Casualties reported amounted to 117, of which 10 ended fatally, 7 being of suicidal intention. The average number of pauper patients relieved during the year was 15,429. These figures are arrived at by taking the total number of days for which relief was given and dividing them by 365. The expenditure amounted to £402,254, as compared with £398,682 for the previous year. The total average cost per patient worked out at £26 1s. 5d. The proportion of registered patients per 100,000 of population on January 1, 1910, was 364. While there has been a remarkable fall in late years in the rate of increase of insanity, there is still an absolute increase in proportion to population. Thus in the quinquenniad 1881-1885, 272 lunatics were resident per 100,000 of population; in 1886-1890 the number was 290, or an increase of 18; in 1891-1895 it was 313, or 23 increase; in 1896-1900 it was 337, or 24 increase; in 1901-1905 it was 357, or 20 increase; while in 1906-1910 it was 364, or an increase of only 7 per 100,000. These figures can, however, only shed an indirect light upon the rate of production of insanity. The Commissioners therefore conclude (1) that the number of patients on the register has increased in the past five years, but that the increase is greatly below that shown by any quinquenniad during the past 25 years. (2) That the occurrence of insanity as shown by admissions to the register in proportion to population shows a marked decrease throughout the past five years. (3) That this decrease is confined to no particular part of the country, but is manifested throughout the country, and irrespective of whether the population of a district is rising or falling. (4) That the number of patients removed from the register by discharge as recovered or unrecovered has been falling continuously during the past thirty years, and never to so large an extent as during the past five years. (5) That the removals from the register by death have risen to some extent, especially during the past ten years. (6) That the increased number of patients found on the register during the past five years is due solely to accumulation caused by a continuous fall in the number of patients discharged, to an extent which has outbalanced the effects of a lowered admission rate and a higher death-rate.

